# Supplementary material for: Soluble P‐tau217 reflects amyloid and tau pathology and mediates the association of amyloid with tau
Source: EMBO Mol Med. 2021 May 5;13(6):e14022. doi: 10.15252/emmm.202114022 (PMC8185545; doi:10.15252/emmm.202114022)
Supplement: Supplementary file 1 — Table EV1 [file EMMM-13-e14022-s001.docx]

**Expanded View Table 1. Neuropathological features of study participants**

| **Article ID** | **Tangle count** | **Braak score** | **Plaque count** | **CERAD** | **Thal Phase** | **Clinical diagnosis** | **Primary neuropathological diagnosis** | **Neuropathological co-pathologies** |
| --- | --- | --- | --- | --- | --- | --- | --- | --- |
| 1 | 15 | V | 15 | frequent | Phase 5 (A3) | Alzheimer's disease | AD | Microscopic changes of Lewy body disease, AGD, infarcts, TDP-43 proteinopathy |
| 2 | 3.5 | III | 0 | zero | Missing/unknown | Parkinson's disease | Parkinson's disease | Definite PART, AGD |
| 3 | 7.5 | IV | 15 | frequent | Phase 5 (A3) | Alzheimer's disease | AD | White matter changes, hemorrhage, infarcts, TDP-43 proteinopathy |
| 4 | 7.5 | IV | 0 | zero | Phase 0 (A0) | Vascular parkinsonism | Vascular dementia | Definite PART |
| 5 | 2.5 | II | 0 | zero | Phase 0 (A0) | Multiple sclerosis | Multiple sclerosis | Definite PART |
| 6 | 3.5 | III | 9 | moderate | Phase 2 (A1) | Parkinson's disease | AD | Parkinson's disease, infarcts |
| 7 | 6.5 | IV | 0 | zero | Phase 0 (A0) | Control | Definite PART | NA |
| 8 | 5 | IV | 0 | zero | Phase 0 (A0) | Parkinson's disease | Parkinson's disease | Definite PART, AGD, ARTAG |
| 9 | 3.5 | II | 7.5 | sparse | Phase 2 (A1) | Parkinson's disease | Parkinson's disease | Possible PART |
| 10 | 7 | IV | 14 | frequent | Missing/unknown | Control | AD pathology, but insufficient for diagnosis | Infarcts |
| 11 | 7.5 | IV | 14 | frequent | Phase 3 (A2) | Dementia NOS | AD | Astrocytoma, cerebral amyloid angiopathy, incidental Lewy body disease, infarcts, radiation changes |
| 12 | 5 | III | 0 | sparse | Phase 0 (A0) | Parkinson's disease | Parkinson's disease | Possible PART, AGD, ARTAG |
| 13 | 7 | IV | 8.5 | frequent | Phase 2 (A1) | Alzheimer's disease | AD | AGD, ARTAG |
| 14 | 15 | V | 15 | frequent | Phase 5 (A3) | Alzheimer's disease | AD | Infarcts, Microscopic changes of Lewy body disease |
| 15 | 4 | III | 0 | zero | Phase 0 (A0) | Control | Definite PART | Cerebral amyloid angiopathy, incidental Lewy body disease |
| 16 | 3 | II | 0 | zero | Phase 0 (A0) | Parkinsonism | White matter changes | Definite PART, infarcts |
| 17 | 0 | I | 0.5 | zero | Phase 1 (A1) | Amyotrophic lateral sclerosis | Amyotrophic lateral sclerosis | Definite PART |
| 18 | 14 | V | 14 | frequent | Phase 5 (A3) | Alzheimer's disease | AD | White matter changes, Alzheimer type II astrocytosis, microscopic changes of Lewy body disease |
| 19 | 6.75 | IV | 12.5 | frequent | Missing/unknown | Control | AD pathology, but insufficient for diagnosis | White matter changes, infarcts, AGD |
| 20 | 6.5 | III | 0 | zero | Phase 0 (A0) | Control | Definite PART | White matter changes, cerebral amyloid angiopathy |
| 21 | 6.5 | III | 0 | zero | Phase 0 (A0) | Parkinson's disease | Parkinson's disease | Definite PART, infarcts |
| 22 | 7 | IV | 13.5 | frequent | Missing/unknown | Mild Cognitive Impairment | AD pathology, but insufficient for diagnosis | White matter changes |
| 23 | 3 | III | 0 | zero | Phase 0 (A0) | Parkinson's disease | Parkinson's disease | Definite PART, cerebral amyloid angiopathy |
| 24 | 7.5 | IV | 13.5 | frequent | Missing/unknown | Alzheimer's disease | AD | Etat crible, white matter changes, focal marked unilateral loss of cervical spinal cord anterior horn motor neurons |
| 25 | 4.5 | IV | 0 | zero | Missing/unknown | Parkinsonism | Definite PART | AGD, infarct |
| 26 | 11.5 | VI | 14.5 | frequent | Phase 4 (A3) | Mixed vascular dementia | AD | Dementia with Lewy bodies, white matter changes, infarcts, ARTAG |
| 27 | 7 | IV | 0.5 | sparse | Phase 1 (A1) | Parkinson's disease | Parkinson's disease | Possible PART, white matter changes |
| 28 | 3.5 | III | 0 | zero | Phase 0 (A0) | Frontotemporal Dementia | FTLD with TDP-43 proteinopathy | Definite PART, microscopic changes of Lewy body disease |
| 29 | 5 | III | 9 | frequent | Phase 3 (A2) | Alzheimer's disease | AD | NA |
| 30 | 9 | IV | 14 | frequent | Phase 5 (A3) | Mild Cognitive Impairment | AD | White matter changes |
| 31 | 10.5 | V | 14.5 | frequent | Phase 5 (A3) | Alzheimer's disease | AD | AGD, ARTAG, microscopic changes of Lewy body disease |
| 32 | 9.5 | V | 14.5 | frequent | Phase 5 (A3) | Alzheimer's disease | AD | White matter changes, moderate to marked Purkinje cell loss, ARTAG |
| 33 | 9.5 | V | 13.5 | frequent | Phase 5 (A3) | Alzheimer's disease | AD | Hippocampal sclerosis, TDP-43 proteinopathy, white matter changes, microscopic changes of Lewy body disease |
| 34 | 7.5 | IV | 1 | sparse | Phase 1 (A1) | Parkinson's disease | Parkinson's disease | Possible PART, ARTAG, AGD |
| 35 | 8 | IV | 0 | zero | Phase 0 (A0) | Mild Cognitive Impairment | None | Definite PART, incidental Lewy body disease |
| 36 | 8 | IV | 4.5 | zero | Phase 2 (A1) | Mild Cognitive Impairment | None | Definite PART, AGD, ARTAG |
| 37 | 8.5 | IV | 13 | frequent | Phase 4 (A3) | Mild Cognitive Impairment | AD | Infarcts, white matter changes |
| 38 | 15 | V | 14.5 | frequent | Phase 5 (A3) | Alzheimer's disease | AD | Severe amyloid angiopathy, white matter changes, microscopic changes of Lewy body disease |
| 39 | 11 | V | 14.5 | frequent | Phase 5 (A3) | Mixed vascular dementia | AD | Severe amyloid angiopathy, white matter changes, hemorrhages, infarcts, cerebellar cortical sclerosis, microscopic changes of Lewy body disease |
| 40 | 10 | V | 15 | frequent | Phase 5 (A3) | Alzheimer's disease | AD | Microscopic changes of Lewy body disease, meningioma |
| 41 | 6.5 | IV | 15 | frequent | Phase 3 (A2) | Parkinson's disease | AD | Parkinson's disease, white matter changes, infarct |
| 42 | 8.5 | IV | 13 | frequent | Phase 3 (A2) | Progressive supranuclear palsy | AD | Progressive supranuclear palsy, white matter changes, infarcts, AGD, ARTAG, microscopic changes of Lewy body disease |
| 43 | 7 | IV | 14.5 | frequent | Phase 5 (A3) | Mixed vascular dementia | AD | White matter changes, infarcts, Wallerian degeneration, left cerebral peduncle of midbrain, AGD |
| 44 | 6 | IV | 0 | zero | Phase 0 (A0) | Control | Definite PART | AGD, ARTAG |
| 45 | 7.5 | IV | 3 | sparse | Phase 1 (A1) | Control | Possible PART | AGD |
| 46 | 7 | V | 13 | frequent | Phase 4 (A3) | Alzheimer's Disease & Vascular dementia | AD | White matter changes, infarcts, ARTAG |
| 47 | 12.5 | VI | 14.5 | frequent | Phase 5 (A3) | Alzheimer's disease | AD | Progressive supranuclear palsy, infarcts, ARTAG |
| 48 | 6.5 | III | 0 | zero | Phase 0 (A0) | Parkinson's disease | Parkinson's disease | Definite PART, ARTAG |
| 49 | 3.5 | III | 14 | frequent | Phase 4 (A3) | Control | AD pathology, but insufficient for diagnosis | NA |
| 50 | 5 | III | 0.5 | sparse | Phase 1 (A1) | vascular cognitive impairment | White matter changes | Possible PART, infarcts |
| 51 | 6.5 | IV | 0 | zero | Phase 0 (A0) | vascular cognitive impairment | White matter changes | Definite PART, infarcts, AGD, ARTAG |
| 52 | 4 | III | 0 | zero | Phase 0 (A0) | Parkinson's disease | Parkinson's disease | Definite PART, ARTAG |
| 53 | 6.5 | III | 0 | zero | Phase 0 (A0) | Control | Definite PART | Acute infarct |
| 54 | 6.5 | IV | 0.5 | sparse | Phase 1 (A1) | Control | Possible PART | ARTAG |
| 55 | 6 | III | 13.5 | frequent | Phase 3 (A2) | Control | AD pathology, but insufficient for diagnosis | Purkinje cell loss, cerebellum |
| 56 | 7 | IV | 0 | zero | Phase 0 (A0) | Mild Cognitive Impairment | Definite PART | White matter changes, ARTAG, AGD |
| 57 | 4.5 | III | 4 | frequent | Missing/unknown | Parkinson's disease | Parkinson's disease | White matter changes, infarcts, ARTAG |
| 58 | 3.5 | III | 0 | sparse | Phase 1 (A1) | Parkinson's disease | Parkinson's disease | Possible PART, ARTAG, infarcts |
| 59 | 3.5 | III | 1 | sparse | Phase 1 (A1) | Primary lateral sclerosis | Possible PART | White matter changes |
| 60 | 5.5 | III | 0 | zero | Phase 0 (A0) | Parkinson's disease | Parkinson's disease | Definite PART, white matter changes, infarcts, ARTAG |
| 61 | 4.5 | III | 11.5 | frequent | Phase 3 (A2) | Mild Cognitive Impairment | Glioblastoma | AD pathology, but insufficient for diagnosis, White matter changes, AGD, ARTAG, infarcts |
| 62 | 4.5 | III | 4.5 | sparse | Phase 3 (A2) | Control | None | Possible PART, incidental Lewy body disease |
| 63 | 4.5 | III | 2 | sparse | Phase 1 (A1) | Control | None | Possible PART, ARTAG |
| 64 | 7 | IV | 12 | frequent | Phase 3 (A2) | Alzheimer's disease | AD | AGD, ARTAG, severe loss of Purkinje cells, superior vermis of cerebellum |
| 65 | 7.5 | IV | 0 | zero | Phase 0 (A0) | Control | None | Definite PART, ARTAG, incidental Lewy body disease, white matter changes |
| 66 | 5.5 | III | 2 | sparse | Phase 2 (A1) | Control | None | Possible PART, ARTAG, microscopic changes of Lewy body disease |
| 67 | 5 | IV | 10.5 | frequent | Phase 3 (A2) | Parkinson's disease | None | None |
| 68 | 6 | IV | 0 | zero | Phase 0 (A0) | Control | None | Definite PART, white matter changes |
| 69 | 4.5 | III | 14.5 | frequent | Phase 4 (A3) | Mild Cognitive Impairment | AD pathology, but insufficient for diagnosis | Infarcts |
| 70 | 3 | III | 0 | zero | Phase 0 (A0) | Dementia NOS | Neurofibrillary tangle-predominant dementia | Definite PART, infarcts |
| 71 | 5.5 | IV | 2.5 | sparse | Phase 1 (A1) | Parkinson's disease | Parkinson's disease | Possible PART, AGD, ARTAG, white matter changes |
| 72 | 10.5 | V | 14 | frequent | Phase 4 (A3) | Alzheimer's Disease & DLB | AD | Dementia with Lewy bodies, ARTAG |
| 73 | 6.5 | IV | 11 | frequent | Phase 3 (A2) | Multiple system atrophy | AD | Multiple system atrophy, white matter changes, AGD |
| 74 | 8 | IV | 9.5 | frequent | Phase 3 (A2) | Mild Cognitive Impairment | AD | White matter changes, infarct, ARTAG |
| 75 | 8 | IV | 10 | frequent | Phase 3 (A2) | Parkinsonism | AD | Dementia with Lewy bodies, AGD, ARTAG |
| 76 | 9.5 | V | 14.5 | frequent | Phase 4 (A3) | Alzheimer's disease | AD | None |
| 77 | 6 | IV | 12 | frequent | Phase 3 (A2) | Mild Cognitive Impairment | AD pathology, but insufficient for diagnosis | None |
| 78 | 6 | III | 14 | frequent | Phase 3 (A2) | Control | AD pathology, but insufficient for diagnosis | Infarcts, Purkinje cell loss, cerebellum |
| 79 | 15 | V | 14 | frequent | Phase 4 (A3) | Alzheimer's disease | AD | Severe amyloid angiopathy, white matter changes, infarcts, microscopic changes of Lewy body disease |
| 80 | 5.5 | IV | 0 | zero | Phase 0 (A0) | Control | Definite PART | Astrocytoma diffuse (grade II), AGD, white matter changes |
| 81 | 11.5 | VI | 15 | frequent | Phase 5 (A3) | Alzheimer's disease | AD | Severe amyloid angiopathy, hemorrhage, AGD |
| 82 | 13.5 | VI | 14 | frequent | Phase 5 (A3) | Alzheimer's disease | AD | White matter changes, microscopic changes of Lewy body disease, TDP-43 proteinopathy |
| 83 | 5 | III | 13.5 | frequent | Phase 2 (A1) | Control | AD | White matter changes, infarcts |
| 84 | 5.5 | IV | 13 | frequent | Phase 4 (A3) | Control | AD pathology, but insufficient for diagnosis | Incidental Lewy Body disease, metastatic adenocarcinoma, infarct, ARTAG |
| 85 | 8.5 | IV | 12.5 | frequent | Phase 3 (A2) | Mixed vascular dementia | AD | TDP-43 proteinopathy, progressive supranuclear palsy, white matter changes |
| 86 | 6.5 | IV | 3 | sparse | Phase 2 (A1) | Control | Possible PART | TDP-43 proteinopathy |
| 87 | 3 | III | 9.5 | frequent | Phase 3 (A2) | Mixed vascular dementia | AD | White matter changes, infarcts |
| 88 | 5 | III | 8 | frequent | Phase 3 (A2) | Parkinson's disease | AD | Parkinson's disease, infarct |

Tangle counts and plaque counts are from global scores which range from 0 to 15. CERAD scores refer to presence and frequency of neuritic plaques.
